# The Epigenetic Effects of Prenatal Cadmium Exposure

**DOI:** 10.1007/s40572-015-0049-9

**Published:** 2015-04-08

**Authors:** Nadia Vilahur, Marie Vahter, Karin Broberg

**Affiliations:** Institute of Environmental Medicine, Unit of Metals and Health, Karolinska Institutet, Nobels väg 13, Box 210, SE-171 77 Stockholm, Sweden

**Keywords:** Cadmium, DNA methylation, DNA methyltransferase, Early life, Metal, Placenta, Prenatal

## Abstract

Prenatal exposure to the highly toxic and common pollutant cadmium has been associated with adverse effects on child health and development. However, the underlying biological mechanisms of cadmium toxicity remain partially unsolved. Epigenetic disruption due to early cadmium exposure has gained attention as a plausible mode of action, since epigenetic signatures respond to environmental stimuli and the fetus undergoes drastic epigenomic rearrangements during embryogenesis. In the current review, we provide a critical examination of the literature addressing prenatal cadmium exposure and epigenetic effects in human, animal, and in vitro studies. We conducted a PubMed search and obtained eight recent studies addressing this topic, focusing almost exclusively on DNA methylation. These studies provide evidence that cadmium alters epigenetic signatures in the DNA of the placenta and of the newborns, and some studies indicated marked sexual differences for cadmium-related DNA methylation changes. Associations between early cadmium exposure and DNA methylation might reflect interference with de novo DNA methyltransferases. More studies, especially those including environmentally relevant doses, are needed to confirm the toxicoepigenomic effects of prenatal cadmium exposure and how that relates to the observed health effects of cadmium in childhood and later life.

## Introduction

### Exposure to Cadmium

Cadmium is ubiquitous in the environment. Exposure to the general population occurs mainly not only via intake of plant-derived foods and certain types of seafood but also through tobacco smoking and industrial emissions [1]. In general, women have higher internal levels of cadmium than men, likely as a result of higher gastrointestinal absorption of cadmium in women with low iron stores or due to genetic factors [2–5]. Further, pregnant women appear to accumulate more cadmium than nonpregnant women [4].

Unlike arsenic, lead, and methylmercury, which effectively cross the placental barrier in humans and other mammals leading to direct fetal exposure [6], cadmium is largely retained in the placenta, where it can accumulate to high concentrations [7, 8, 9••].

### Cadmium Toxicity During Critical Developmental Periods

Cadmium is a highly toxic metal ranked among the top five on the 2011 Agency for Toxic Substances and Disease Registry (ATDSR) Priority List of Hazardous Substances [10]. Numerous studies in adults have reported that cadmium exposure has adverse effects on the kidney and bone; more recently, low-dose cadmium intake has been associated with the risk of developing cardiovascular disease and some types of cancer [1, 11–13]. There is increasing evidence that cadmium can also be toxic early in life, with effects that may persist over time. Epidemiological studies have reported associations between prenatal cadmium exposure, measured through concentrations in maternal urine or cord blood, and negative effects on anthropometric outcomes in newborns and children at 3–5 years of age, with some studies pointing towards stronger associations in girls compared to boys [14, 15••, 16–18]. Moreover, increased concentrations of cadmium have been found in placentas from mothers delivering low birth weight neonates [19]. There is some evidence indicating that prenatal exposure to cadmium may affect the immune system from a very early age, expressed as a higher risk of atopic dermatitis in 6-month-old infants [20]. Mice prenatally exposed to environmentally relevant doses of cadmium showed postnatal alterations in immune cell development and function [21].

Cadmium can act as an endocrine disrupting chemical, since it has been shown to interfere with progesterone, testosterone, and leptin synthesis and to alter the thyroid hormone system [22–25]. Mice and rats prenatally exposed to cadmium showed impairments of their reproductive systems, both in males and in females [26, 27].

The mechanisms of early life toxicity of cadmium are not yet clear. Cadmium exposure during pregnancy, leading to accumulation in the placenta, seems to disturb zinc transfer to the fetus, interfere with glucocorticoid balance, and affect the regulation of insulin growth factor-related proteins; and it has also been shown to inhibit the migration of human placental trophoblast cells [24, 28, 29, 30••, 31, 32]. Additionally, low-level exposure to cadmium has been associated with increased oxidative stress in early life [33]. A possible underlying molecular mechanism for the prenatal action of cadmium is through interference with the epigenetic machinery.

### Early Environmental Exposures and Epigenetics

In the last decade, epigenetic processes have gained attention as potential mediators of the adverse health effects observed in relation to exposure to environmental toxicants, an expanding area of research known as *toxicoepigenomics* [34–36]. Epigenetic marks, broadly defined as dynamic changes to the genome other than changes in the DNA sequence itself, can affect the regulation of gene expression and can be transmitted through mitosis and/or possibly through meiosis [37]. These epigenetic marks include methylation on the cytosine residues of DNA (when the cytosine is followed by a guanine, a CpG site), posttranslational modifications of histone proteins, and small noncoding RNA molecules (so-called microRNAs) that may interfere with gene transcription and/or translation [38–41]. Epigenetic mechanisms have key functions in regulating cellular homeostasis, lineage-specific gene expression, and establishment of developmental programs, which may have long-lasting influence on future health. During human embryonic development, a series of epigenetic changes take place at the zygote stage and during formation of primordial germ cells; these changes involve two main waves of genome-wide epigenomic reprogramming leading to the sequential activation and repression of genes important for the control of growth and metabolism [42–44]. In addition, sex-specific differences in epigenetic marks are generated early after fertilization [45].

This window of epigenetic developmental plasticity in humans, from preconception to early childhood, represents a period of enhanced vulnerability to environmental stimuli that can lead to aberrant gene expression, enhance the risk of disease in early life, and program individuals for risks of disease in adult life [46]. Indeed, epigenetic effects early in life have been found for prenatal toxic exposures such as arsenic and tobacco smoke [47–49].

The aim of the present review was to summarize the current state of knowledge and provide a critical examination of reported epigenetic changes caused by in utero exposure to cadmium. To that end, we have selected and summarized original research articles that have addressed this topic in epidemiological, animal, and in vitro studies conducted on relevant cell types.

## Methods

### Sources of Information and Search Strategy

We conducted a bibliographic search using the electronic data source PubMed (National Library of Medicine, Bethesda, MD, USA: http://www.ncbi.nlm.nih.gov/pubmed), and included in this review all published articles in English within a time frame of 10 years (31st December 2004 to 31st December 2014) that reported any epigenetic data measured either in blood or in other tissues in relation to prenatal exposure to cadmium in humans or animal models. In addition, we also included in vitro studies on specific cell types relevant to prenatal exposure, such as embryonic stem cells, fetal-derived cells, or placental trophoblasts. The references of the selected articles were also used as an additional source of relevant studies.

The following key terms and their combinations were used in the literature search: “cadmium”, “prenatal”, “pregnancy”, “maternal”, “epigenetic”, “methylation”, “histone”, “microRNA”, “in utero”, and “metal*”.

## Results

### Literature Review

A total of eight original research articles were identified according to our search criteria. Among them, three were human cohort studies, two were conducted in animals, and three were in vitro. The characteristics and main results of these studies are summarized in Tables 1, 2, and 3.

**Table 1:** Main characteristics of the studies (all three were cohort studies) conducted in humans analyzing associations between prenatal exposure to cadmium and epigenetic changes

First author, year	Population; *n*	Prenatal cadmium exposure, concentration (range)	Epigenetic modification	Tissue/s	Expression changes	Main outcomes in relation to cadmium exposure
Boeke CE, 2012	Massachusetts, USA; *n* = 557 mother-child pairs	Estimated by FFQ (T1), AM: 15.38 μg/day (14.7–16.3)	Global DNA methylation (LINE-1 methylation)	Maternal blood (T1 and T3), cord blood	Na	Hypermethylation in maternal DNA (T1), hypomethylation in fetal DNA
Kippler M, 2013	Matlab, Bangladesh; *n* = 127 mother-child pairs	Urine (GW8), M 0.77 μg/L (0.25–2.4); Blood (GW14), M 1.3 μg/kg (0.54–3.1)	Genome-wide DNA methylation (450 K array)	Cord blood, children blood (4.5 years)	Na	Sex-specific correlations with maternal blood cadmium: in boys, 96 % of 500 top CpG sites hypermethylated; in girls, 21 % hypermethylated
Sanders AP, 2014	North Carolina, USA; *n* = 17 mother-child pairs	Blood (T3); AM: 0.44 μg/L (0–1.05)	Genome-wide DNA methylation (MIRA, Affimetrix array)	Maternal blood (T3), cord blood	Na	Maternal DNA: hypermethylation in 81 genes hypomethylation in 11 genes fetal DNA: hypermethylation in 90 genes hypomethylation in 1 gene

*T1*/*T3* first/third trimester of pregnancy, *M* median, *AM* arithmetic mean, *GW* gestational week, *FFQ* food frequency questionnaire, *LINE*-*1* long interspersed nuclear element-1, *450 K* Illumina Infinium Human Methylation 450, *MIRA* methylated CpG island recovery assay; expression changes refer to the level of mRNA and/or protein

**Table 2:** Main characteristics of the studies in animals analyzing associations between prenatal exposure to cadmium and epigenetic changes

First author, year	Model	Number	Prenatal cadmium exposure: length; dose	Exposure timing	Epigenetic modification	Tissue	Expression changes	Main outcomes in relation to cadmium exposure
Doi T, 2011	Chicks (Ross strain)	*n* = 39 (13 each time point)	During 1, 4, and 8 h; 50 μl at 50 μM	Embryos at Hamilton Hamburger stages 16 or 17 (60-h postfertilization)	Global DNA methylation (total methylcytidine content)	Whole organism	Yes	After 4 h of exposure (64-h postfertilization), global hypomethylation and lower expression of *DNMT3A* and *DNMT3*B. No effects after 8 h (68-h postfertilization)
Castillo P, 2012	Wistar rats	*n* = 24 (12 cases/12 controls)	From weaning until mating; 10 ppm	Pre-mating and prenatal (whole pregnancy)	Gene-specific DNA methylation (bisulphite pyrosequencing)	Liver	Yes	Females: hypomethylation of GR, increased expression of GR (mRNA and protein) and lower *DNMT3A* expression; males: opposite effects
			From mating until day 20 of gestation; 50 ppm (ad libitum)					

*DNMT* DNA methyltransferase, *GR* glucocorticoid receptor; expression changes refer to the level of mRNA and/or protein

**Table 3:** Main characteristics of in vitro studies analyzing associations between cadmium exposure and epigenetic changes

First author, Year	Cell type	Cadmium exposure: duration; dose (range)	Epigenetic modification	Expression changes	Main outcomes in relation to cadmium exposure
Jiang G, 2008	Human embryo lung fibroblasts (HLF)	2 months; 0–1.5 μmol/L	Global DNA methylation (HPLC)	Yes	Global hypermethylation, overexpression of *DNMT1*, *DNMT3A*, and *DNMT3B*
Ronco AM, 2010	Human choriocarcinoma cells (JEG-3)	24 h; 0.5–1 μM	Gene-specific DNA methylation (methylation-specific PCR)	Yes	Lower *HSD11B2* methylation, overexpression of 11β-HSD2
Gadhia SR, 2012	Mouse embryonic stem cells (mES)	1 and 24 h; IC_25_ (0.08 mM) and IC_50_ (0.16 mM)	Global histone H3K27 monomethylation (fluorimetric assay)	Yes	H3K27-me1 hypomethylation after 24-h exposure at IC_50_; no changes in expression after 1-h exposure

*IC*_*25*_ and *IC*_*50*_ inhibitory concentration 25 and 50 % derived from MTT cytotoxicity experiments, *HPLC* reversed-phase high-pressure liquid chromatography, *H3K27*-*me1* histone 3-lysine27 monomethylation; expression changes refer to the level of mRNA and/or protein

### Human Studies

Three recent epidemiologic studies explored the association between prenatal cadmium exposure and DNA methylation in blood cells (Table 1). One study measured global DNA methylation using as a proxy the methylation of the human long interspersed nuclear element 1 (LINE-1), a transposable repetitive sequence that constitutes approximately 17 % of the human genome [50]. LINE-1 is typically highly methylated in normal tissues, whereas hypomethylation of LINE-1 is commonly observed in cancerous tissues and is believed to cause alterations in gene expression and chromatin packaging, as well as increasing genomic instability [51–53]. Here, periconceptional cadmium intake, estimated from food frequency questionnaires (expressed per increase in one standard deviation or ∼4 μg Cd/day), was significantly associated with LINE-1 hypomethylation in cord blood DNA (*n* = 557 newborns) in fully adjusted models including methyl donors (vitamin B_12_, betaine, choline, and folate), child’s sex, maternal age, race, smoking, gestational weight gain, and education as covariates [50]. Cadmium-related hypomethylation of LINE-1 could increase its transposase activity, which may induce genomic alterations that could be associated with adverse chronic health outcomes later in life [54, 55]. In the same study, the authors also found that the estimated cadmium intake was associated with LINE-1 DNA hypermethylation in maternal blood in the first trimester of pregnancy, a finding that is in contrast to a study in adult nonpregnant and nonsmoking Andean women in Argentina, where low-level cadmium exposure was associated with LINE-1 hypomethylation in peripheral blood DNA [56].

The two other studies measured genome-wide DNA methylation using commercial DNA methylation arrays that screen a large number of individual CpG sites throughout the genome. The first is a cohort study based on 127 mother-child pairs from a rural area of Bangladesh. All participant mothers were nonsmokers, and cadmium exposure, mostly derived from food consumption, was based on measured concentrations in maternal blood collected in early pregnancy around week 14 (median 1.3, range 0.38–5.4 μg/kg) [57]. DNA methylation was assessed in cord blood using the Illumina HumanMethylation450K array, which measures approximately 450,000 CpG sites. No significant associations of cadmium and DNA methylation were found after adjustments for multiple testing. However, a marked sex dimorphism in cadmium-related methylation changes was observed, which seemed to be more pronounced in boys: 96 % of the top 500 CpGs in boys showed hypermethylation in relation to increasing cadmium in maternal blood, while only 29 % of the top CpGs were hypermethylated in girls. Neither verification nor validation of the top cadmium-associated CpG sites using another technique, e.g., bisulfite pyrosequencing was conducted in this study. Pathway analysis, where cadmium-related CpGs are annotated to biological function, showed some overlapping pathways for both sexes; and moreover, cell death-related genes were enriched in boys, whereas embryonic and organ development-related genes were enriched in girls. Finally, among the 500 top CpGs positively correlated with cadmium, four (three in girls and one in boys) were also associated with lower birth weight in covariate-adjusted models (although associations did not reach significance after multiple-testing correction), thus suggesting that DNA methylation may be involved in the cadmium-related impairment of fetal growth.

The second study examined 17 mother-child pairs and was nested in a larger study of a population from the USA. Cadmium concentrations were measured in blood collected at delivery, and maternal-newborn pairs were divided into two exposure categories (high or low) based on maternal blood cadmium 50th percentile for pregnant women (0.2 μg/L) according to the National Health and Nutrition Examination Survey (NHANES) [58]. DNA methylation was measured using the Methylated CpG island recovery assay (MIRA) followed by hybridization to the Affymetrix Human Promoter 1.0R arrays (Affymetrix), which assesses over 4.6 million sites located in human gene promoter regions. Non-overlapping gene sets were found in maternal (*n* = 92 genes) and in fetal (*n* = 61) blood DNA that were differentially methylated (defined as an average island promoter methylation with a minimum absolute change of 30 %; and a *P* value <0.05 measured by ANOVA) in relation to prenatal cadmium exposure, and these were always hypermethylated, although they were not statistically significant after false discovery rate adjustment. Similarities at the biological pathway level were identified between the two groups of genes (maternal vs. fetal) with an enrichment of genes that play a role in transcriptional regulation and apoptosis. The same study evaluated DNA methylation changes in relation to cotinine, a primary biomarker of nicotine exposure, measured in maternal serum and dichotomized as presence or absence according to the detection limit. A total of 366 cotinine-associated differentially methylated genes were found in fetal DNA, and there was an overlap of 30 genes between the cadmium and the cotinine-associated gene sets, which were hypermethylated in relation to both exposures. Finally, the methylation degree of the apoptosis-related gene *PRR13*, identified as differentially methylated only in fetal DNA in relation to cadmium using the MIRA assay, was verified in DNA of 15 newborns by methylation-specific quantitative polymerase chain reaction, and both techniques were found to be in relatively good agreement (Spearman’s rank correlation *r* = 0.53, *P* < 0.05). No validation of results was conducted in additional samples.

The findings of the two genome-wide studies are difficult to compare, mainly because of differences in study design including exposure assessment timing (first trimester vs delivery), genome-wide coverage of CpG sites and sample size, which was too small in the study by Sanders and collaborators to stratify by sex. Notably, both studies showed changes in methylation in genes related to cell death with more cadmium exposure; effects on these genes could be linked to different health effects of cadmium, including cancer [59, 60]. Finally, none of the studies conducted in cord blood DNA were able to perform cell sorting or conducted statistical adjustments for cell composition estimated using methods such as the Houseman, recently adapted as well for tissues for which reference datasets are unavailable [61]; and this might represent a source of unmeasured confounding and, more importantly, underpower the estimation of cadmium-induced epigenetic effects.

### Animal Studies

Different tissues, including term placenta, cord blood, and saliva in newborns have unique epigenetic signatures, both globally and in specific loci [62]. Animal studies therefore represent a valuable tool to study epigenetic effects of exposure to toxic chemicals during pregnancy on organs that otherwise would remain difficult to explore in humans, especially in newborns and children.

In a study performed on chick embryos, where a single dose of 50 μL of 50 μM (5.620 μg/L) cadmium (i.e., 0.281 ng of cadmium) was administered and compared to control embryos administered with saline only, decreased global DNA methylation was reported during very early embryogenesis 64 h after fertilization (4-h post-cadmium treatment), measured in situ in whole embryos using immunohistochemical staining with a monoclonal antibody against 5-methylcytidine. These changes in DNA methylation were accompanied by lower levels of expression of the de novo methyltransferases *DNMT3A* and *DNMT3B* at this specific stage [63]. None of these effects were observed for cadmium exposure at 68 h after fertilization (8-h post-cadmium treatment). The authors suggested that the epigenetic changes early in embryogenesis could be related to defects in abdominal wall development in the chick embryos as a result of cadmium exposure, previously reported by the same laboratory [64]. Another study evaluated the effect of cadmium exposure through drinking water at 50 ppm (50.000 μg/L) administered ad libitum to rats during the whole pregnancy on DNA methylation of the glucocorticoid receptor in the liver of the offspring, measured by bisulfite pyrosequencing [65]. Here, male and female offspring (*n* = 6 in each group) were analyzed separately at the end of pregnancy (day 20), and opposite effects were observed between the sexes in relation to this high level of cadmium exposure (estimated 5 ng per 100 mL of drinking water): males showed glucocorticoid receptor hypermethylation accompanied by lower gene expression, while females showed hypomethylation and overexpression of the gene, when compared to non-exposed controls of the same sex. In addition, the cadmium-induced changes in DNA methylation correlated with higher levels of expression of *DNMT3A* methyltransferase in male and lower levels in female rats. The sex-specific changes in DNA methylation, i.e., hypermethylation in males and hypomethylation in females, are in line with the results found in human newborns exposed to much lower cadmium concentrations [57].

### In Vitro Studies

In the only study measuring histone marks, mouse embryonic stem cells were exposed to cadmium chloride (CdCl_2_) for 1 and 24 h at concentrations of IC_25_ and IC_50_ (extrapolated from concentration–effect curves; equivalent to a cadmium concentration of 0.08 and 0.16 mM, or 9000 and 16.000 μg/L, respectively) [66]. A significant reduction (more than 50 %) of global histone lysine monomethylation (H3K27me1), measured by a fluorimetric assay, was observed in cells after 24 h of exposure at IC_50_, when compared to non-exposed cells, and although nonsignificant, a decrease in methylation of around 20 % was already visible at IC_25_. The reduction in histone methylation due to cadmium was similar to that observed for equivalently high concentrations of arsenic, mercury, and nickel in the same study. Since H3K27me1 is linked to activation of transcription, these results suggest that cadmium could trigger transcriptional repression. The effect of long-term and relatively low-dose exposure to cadmium on global DNA methylation was analyzed in human embryo lung fibroblasts by measuring the total content of methylated cytosine using reversed-phase high-pressure liquid chromatography [67]. A significant dose-dependent increase of 26.9 and 31.4 % in global methylation was observed in cells exposed for 2 months to cadmium at concentrations of 1.2 and 1.5 μmol/L (135 and 169 μg/L), respectively, when compared to controls. This hypermethylation was accompanied by an upregulation in the expression of DNA methyltransferases *DNMT1*, *DNMT3A*, and *DNMT3B*, together with an increase in cell growth. Finally, acute cadmium exposure in cultured human choriocarcinoma cells (JEG-3), i.e., cells derived from a trophoblastic cell carcinoma, was shown to affect expression of the 11b-hydroxysteroid dehydrogenase type 2 enzyme (11b-HSD2; encoded by *HSD11B2*), which is one of the most important regulators of levels of glucocorticoid in placental tissue [68]. Cadmium exposure at a dose of 1 μM (112 μg/L) for 24 h was associated with a significantly higher *HSD11B2* expression, a concomitant (although nonsignificant) reduction in the promoter DNA methylation of *HSD11B2* measured by methylation-specific PCR, and a lower cortisol production [69]. This is in contrast to the results of Yang et al. [31], showing a concentration-dependent decrease in expression of *HSD11B2* with a maximal effect at 0.75 μM (84 μg/L), accompanied by higher levels of cortisone in normal trophoblast cells exposed to cadmium in the same conditions. Here, no measures of DNA methylation were conducted. However, caution should be taken in comparing the results of the two studies on cadmium and *HSD11B2*, since the JEG-3 choriocarcinoma cells are tumoral in origin and may behave differently than normal trophoblasts in their epigenetic regulation.

## Discussion and Conclusions

The body of literature regarding prenatal exposure to cadmium and epigenetic alterations was found to be scarce. However, all the existing studies ranging from low to very high doses of exposure reported epigenetic changes in relation to cadmium, pointing towards epigenetic dysregulation as a mechanism for early life cadmium toxicity. In particular, associations of cadmium with DNA methylation of certain CpG sites within genes of interest in organ development, glucocorticoid synthesis, and cell death have been reported. It remains to be shown whether the interference of cadmium with epigenetic processes occurs by direct action in the embryo or fetus or indirectly via effects on the placenta. Whatever mechanism, this may result in aberrant gene expression in different tissues of the developing child, which could explain the various adverse outcomes associated with prenatal cadmium exposure, i.e., impaired growth, immune toxicity, and endocrine disruption.

Two studies, one on low-dose exposed women and the other on extremely highly dosed rats, reported that cadmium exposure in pregnancy alters fetal DNA methylation in a marked sex-specific manner [57, 65], which may help clarify previous findings of differences in toxicity of cadmium observed between girls and boys [14, 15••, 16]. Sex-specific epigenetic changes have been reported in relation to other environmental pollutants as well, such as arsenic and endocrine disrupting chemicals [47, 70, 71], and stresses the need to evaluate the influence of sex in future toxicoepigenomic studies.

A further aspect important to consider for these studies and their comparison is that cadmium-related epigenetic effects might differ upon low (environmentally relevant) and high (likely toxic) doses of exposure. Although epigenetic effects might only arise upon reaching a certain concentration, as it has been reported in relation to cadmium and gene expression [31, 69], a major problem with several of the experimental studies is the very high doses used, which might not be applicable to cadmium exposure in human populations. This is an important consideration, especially if epigenetic data in the future should be incorporated in risk assessment processes for toxic metals, as discussed in detail by Ray and coworkers [36].

Cadmium-induced changes in levels of DNA methyltransferases could be one mechanism underlying the epigenetic alterations caused by cadmium, although the direction of the effects remains unclear. Three of the studies reviewed in the present work reported changes both in DNA methylation and in expression of DNA methyltransferases in relation to prenatal cadmium exposure. Two of them, conducted in animals at very high exposure levels, found a decrease in expression of de novo DNA methyltransferases (both *DNMT3A* and *DNMT3B* or only *DNMT3A*) along with DNA hypomethylation, either global or gene-specific [63, 65]. This is similar to what was reported in a study in adult women exposed to low cadmium levels from normal food (median 0.23 μg/L in urine), where increasing cadmium concentrations were associated with LINE-1 hypomethylation and lower *DNMT3B* expression in peripheral blood [56]. By contrast, opposite results were observed in vitro in human lung embryo fibroblasts, showing higher global DNA methylation and *DNMT1*, *DNMT3A*, and *DNMT3B* expression at concentrations of 135 and 169 μg/L [67], which was consistent with a previous study conducted in human prostate epithelial cells reporting higher methylation and *DNMT3B* expression after long-term, i.e., 10 weeks high dose (1124 μg/L) cadmium exposure [72]. These differences in cadmium and DNA methyltransferases might reflect that cadmium affects these enzymes differently in whole organisms compared to long-term cultured cells or result from the high variation in the doses from study to study.

DNA methylation was the most widely studied epigenetic mark; only one study examined histone modifications and none examined microRNAs. This is likely due to the covalent and more stable nature of DNA methylation compared to other epigenetic signatures [73], together with the availability of techniques that allow its measurement with relatively high-throughput and quantitative technologies. Future studies in this field are recommended to work towards the simultaneous measurement of several epigenetic marks, since they appear to interact in the genomic context [74], and also examine newly described epigenetic signals such as DNA hydroxymethylation, which is thought to regulate gene expression and prompt DNA demethylation in early pregnancy [75, 76]. Integrating different regulatory epigenetic marks will help us gain a better biological picture of the mechanisms related to cadmium toxicity.

It is key in environmental epigenetic studies to choose carefully the target tissue that will be studied. This can be driven by previous knowledge associated either with the exposure, i.e., toxicokinetics and toxicodynamics, with the role of the tissue or the organ in the context of the phenotype studied, or ideally a combination of both. Placenta warrants further attention in human studies of prenatal cadmium effects, considering its multiple biological functions during fetal development, and especially because it has been shown to be a partial barrier to the transfer of cadmium from maternal to fetal blood stream [77].

In conclusion, most of the currently available literature suggests that prenatal exposure to both low and extremely high doses of cadmium is associated with epigenetic effects, representing a plausible mechanism for cadmium toxicity. Although suggestive, the evidence remains too limited to draw firm conclusions on the direction of the epigenetic changes and their direct implication for disease outcomes. In particular, it is difficult to draw conclusions as to the type and degree of disturbances due to the extremely high doses used in several of the animal and in vitro studies. More research in this area is warranted; focusing on environmentally relevant doses and also considering the effects of co-exposure during pregnancy to multiple common environmental contaminants that affect epigenetic marks. Additionally, human studies should aim at larger sample sizes and the most significant results validated in independent but similar populations, especially in the context of epigenome-wide association studies where a large number of CpG sites are evaluated simultaneously [78]. This growing field of research has potential implications for public health in terms of prevention, especially if epigenetic changes due to early life cadmium exposure could be used as predictors of later disease outcome and modified during postnatal life. Therefore, it is important that existing cohort studies conduct follow-up studies, because some of the health effects due to early epigenetic dysregulation from cadmium exposure may likely not arise until later in life, i.e., in adolescence or adulthood.
